# Green synthesis of silver nanoparticles derived from algae and their larvicidal properties to control *Aedes aegypti*

**DOI:** 10.3762/bjnano.15.123

**Published:** 2024-12-04

**Authors:** Matheus Alves Siqueira de Assunção, Douglas Dourado, Daiane Rodrigues dos Santos, Gabriel Bezerra Faierstein, Mara Elga Medeiros Braga, Severino Alves Junior, Rosângela Maria Rodrigues Barbosa, Herminio José Cipriano de Sousa, Fábio Rocha Formiga

**Affiliations:** 1 Aggeu Magalhães Institute (IAM), Oswaldo Cruz Foundation (FIOCRUZ), 50670-420, Recife, PE, Brazil; 2 Chemical Process Engineering and Forest Products Research Centre (CIEPQPF), Department of Chemical Engineering, University of Coimbra, 3030-790, Coimbra, Portugal; 3 Department of Fundamental Chemistry (DQF), Federal University of Pernambuco (UFPE), 50740-560, Recife, PE, Brazil; 4 Faculty of Medical Sciences, University of Pernambuco (UPE), 52171-011, Recife, PE, Brazil

**Keywords:** bioassay, inorganic nanoparticles, mosquito vector, nanotechnology, physicochemical, tropical neglected diseases

## Abstract

Mosquito vectors such as *Aedes spp*. are responsible for the transmission of arboviruses that have a major impact on public health. Therefore, it is necessary to search for ways to control these insects, avoiding the use of conventional chemical insecticides that are proven to be toxic to nature. In the last years, there has been growing evidence for the potential of silver nanoparticles (AgNPs) to be ecologically benign alternatives to the commercially available chemical insecticides against vector-borne diseases. Natural seaweed extracts contain metabolites such as polyphenols, terpenoids, and alkaloids. These compounds act as reducing agents and stabilizers to synthesize biogenic AgNPs. The green synthesis of AgNPs has advantages over other methods, such as low cost and sustainable biosynthesis. In the perspective of using AgNPs in the development of novel insecticides for vector control, this review deals with the eco-friendly synthesis of AgNPs through seaweed extracts as reducing and stabilizing agents. In addition, assessment of toxicity of these nanomaterials in non-target species is discussed.

## Introduction

Arboviroses are diseases caused by the pathogens transmitted by arthropods, and their transmission to humans occurs through the bite of hematophagous arthropods. Mosquitoes are the most important vectors of arboviroses [1], although many are maintained by ticks [2], phlebotomines [3], and other arthropods [4]. Arboviroses represent a major public health concern in tropical and sub-tropical regions of the world [5]. *Aedes aegypti* (Stegomyia) Linnaeus (1762) (Diptera: Culicidae), known as the dengue mosquito, is a vector of important arboviroses, including Dengue, Zika, Chikungunya, and Yellow Fever [6].

Since there are no specific antiviral treatments for arboviruses and the endemicity of these diseases is determined by the presence of the vector, approaches for the control of arthropod-borne diseases involve strategies focused on the vector. These may include the application of synthetic insecticides or the implementation of treatments targeted at patients [7–8]. An emerging strategy for controlling arboviral vectors are nanomaterials or nanomaterial-based formulations as so-called nanopesticides, providing new, modern, and low-cost formulations [9–10] with the ability to penetrate through the exoskeleton into mosquito cells, causing mortality after binding to proteins or DNA [11]. Nanomaterials provide characteristics such as greater absorption capacity, greater bioavailability, controlled release of active ingredients, improved solubility of hydrophobic substances in water, and good kinetic stability [12–14].

Metallic nanoparticles have been investigated as a promising approach for vector control. The chemical reduction of metal ions through biological compounds can be used to synthesize non-toxic and environmentally safe “green” insecticide alternatives in the form of metal-based nanoparticles [15]. A promising option are silver nanoparticles (AgNPs) obtained through synthesis from natural extracts containing secondary metabolites that act as reducing and stabilizing agents. Among these metabolites, alkanes, aromatics, phenols, ethers, amines, and amides stand out for their role in the reduction, stabilization, and capping of silver nanoparticles [11,16–19]. Compounds of natural origin are generally preferred in vector control because of a less deleterious effect on non-target organisms and their inherent biodegradability. The development of sustainable pest control tools is a challenge for researchers and public health authorities [20]. Seaweed extracts are composed of bioactive agents such as phenols, ascorbic acid, flavonoids, polyphenolics, alkaloids, and terpenes, which could act as reducing agents [21].

This review focuses on AgNPs produced in a green and sustainable way through the use of natural products as reducing agents, namely seaweed extracts. The activity of AgNPs upon *A. aegypti* and their potential role for the control and prevention of arboviruses are presented. Finally, ecotoxicity and environmental risk assessment of AgNPs are further discussed.

## Review

### Synthesis of silver nanoparticles

**Figure 1:**
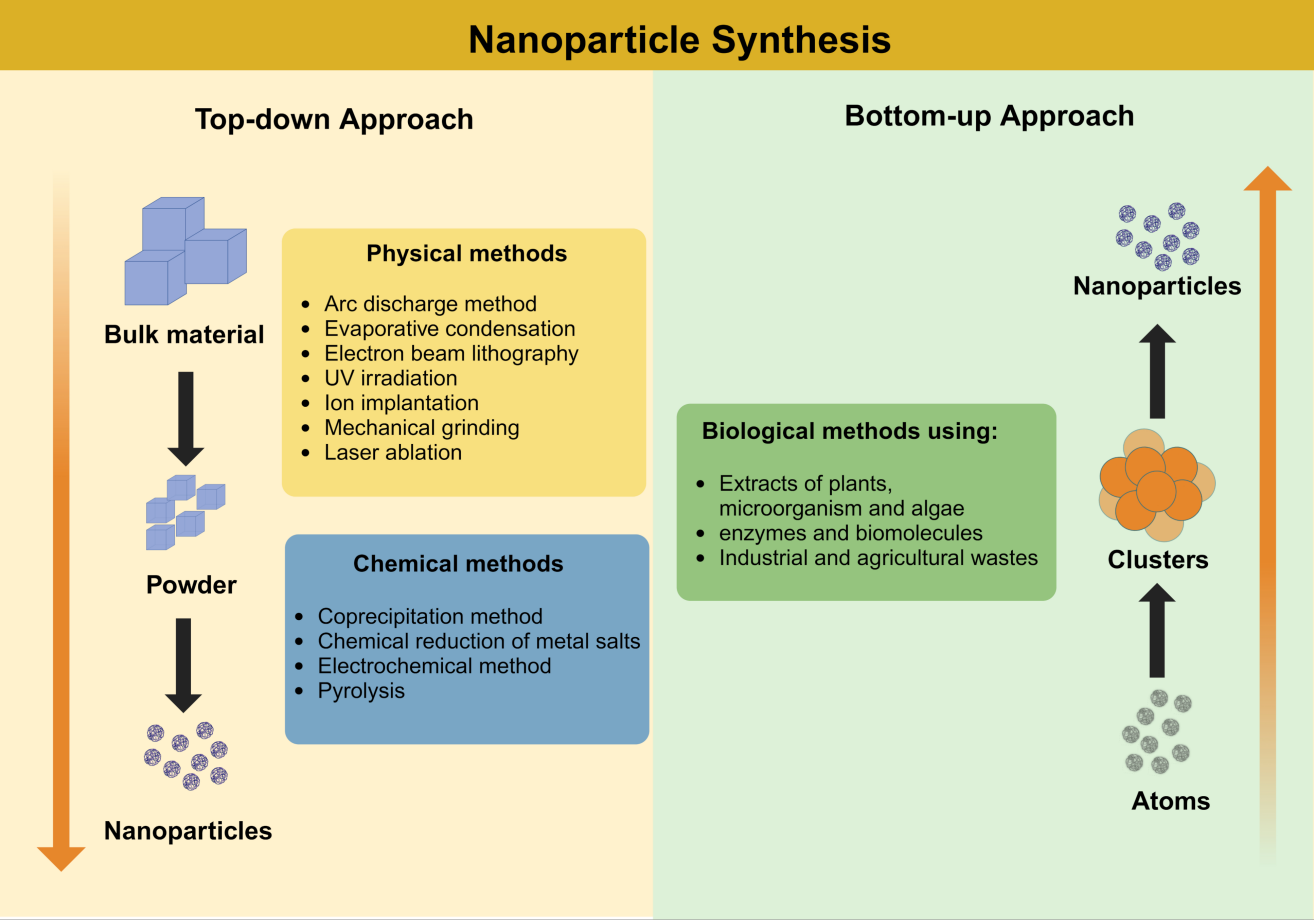
“Top-down” and “Bottom-up” approaches for synthesis of silver nanoparticles. Created in BioRender. Rocha Formiga, F. (2024) https://BioRender.com/a60t035. This content is not subject to CC BY 4.0.

AgNPs are metallic nanoparticles in a size range between 1 and 100 nm with unique electrical, optical, and magnetic properties for a wide range of applications [22–23]. They can be synthesized by different procedures based on “top-down” or “bottom-up” approaches [24] (Figure 1). Top-down synthesized silver nanoparticles can be obtained by lithography, attrition, milling, and other processes that involve reducing the size of bulk silver materials to the atomic size of the AgNPs [25]. Bottom-up AgNPs are synthesized via precursor salt reactions that lead to the formation of AgNPs [26] including condensation, precipitation, and pyrolysis [27].

AgNPs can be synthesized using physical, chemical, or biological methods [28]. Chemical AgNPs synthesis can require toxic substances such as polyvinylpyrrolidone, polyvinyl alcohol, and polyacrylonitrile as stabilizing agents and sodium borohydride, hydrazine, and hydroxylamine as reducing agents [29]. These may generate more toxic chemical residues in the environment [30]. Physical methods include laser ablation, UV irradiation, evaporation condensation, aerosol methods, and lithography. High cost, high energy consumption, and expensive equipment make these techniques uneconomical [31].

Because of these disadvantages, synthesis methods based on naturally occurring biomaterials have been used as an alternative to obtain metallic nanoparticles [32–33]. These do not involve any toxic chemicals and require less energy and synthesis time. Simple protocols have been used involving the reduction of metal ions using biological extracts as reducing agent [34]. In this way, the green synthesis of nanoparticles has expanded in nanoscience and nanotechnology [35].

### Synthesis of silver nanoparticles using algae

Green nanoparticle synthesis is the design and development of strategies for the production of nanoparticles to reduce the use or formation of substances harmful to human health and the environment [36–37]. It has many advantages compared to chemical and physical methods, that is, it is non-toxic, pollution-free, ecological and economical, and more sustainable [38–39]. There is a variety of natural resources for the green synthesis of silver nanoparticles (yeasts, plants, fungi, algae, and bacteria), which are capable of reducing inorganic metal ions to metallic nanoparticles quickly [40–41]. Among these, algae have been highlighted because of their immense bioactive potential of compounds such as accessory pigments, proteins, sulfated polysaccharides and other biomolecules. The latter include flavonoids, alkaloids, steroids, phenols, and saponins with hydroxy, carboxyl, and amino functional groups, which are effective agents in metal reduction and also provide a robust coating on the metallic nanoparticles in a single step [42–46].

These bioactive compounds associated with metallic nanoparticles increase the specific delivery of drugs to the target and, thus, reduce the required amount of active compounds [47]. In addition, the control of particle size and morphology is essential for applications in biotechnology, and the biological approach has the ability to better control the particle size than chemical and physical synthesis methods of metallic NPs [48–49]. Thus, different species of algae have been used in the green synthesis of silver nanoparticles. In this review, species of brown algae (*Sargassum polycystum*, *Sargassum natans*, *Padina gymnospora*), red algae (*Hypnea musciformis*, *Centroceras clavulatum*, *Amphiroa rígida*, *Gracilaria firma*), blue algae (*Oscillatoria sancta*), and green algae (*Ulva lactuta*) are reported as biomass for the green synthesis of AgNPs (Table 1).

**Table 1:** Data from studies on the green synthesis of silver nanoparticles.

Algae extract	Synthesis conditions	Particle characteristics	Reference

aqueous extract of *Sargassum polycystum*	AgNO_3_ concentration – 1 mM reaction period – 3 h reaction temperature – 37–80 °C	SPR^a^ – 418 nm size – 20–88 nm shape – cubical	[51]
ethanol extract of *Hypnea musciformis*	AgNO_3_ concentration – 1 mM reaction period – 120 min reaction temperature – room temperature	SPR^a^ – 420 nm size – 40–65 nm shape – spherical	[52]
ethyl alcohol extract of *Sargassum natans*	AgNO_3_ concentration – 100 mM reaction period – 24 h reaction temperature – room temperature	SPR^a^ – 340 nm size – 50 nm shape – ND^b^	[53]
aqueous extract of *Centroceras clavulatum*	AgNO_3_ concentration – 1 mM reaction period – ND^b^ reaction temperature – room temperature	SPR^a^ – 410 nm size – 35–65 nm shape – spherical and cubic	[50]
aqueous extract of *Amphiroa rigida*	AgNO_3_ concentration – 1 mM reaction period – 30 min reaction temperature – 37 °C	SPR^a^ – 420 nm size – 20–30 nm shape – spherical	[54]
aqueous extract of *Oscillatoria sancta*	AgNO_3_ concentration – 1 mM reaction period – 60 min reaction temperature – 28 °C	SPR^a^ – 450 nm size – 25–50 nm shape – cubical and hexagonal	[55]
aqueous extract of *Gracilaria firma*	AgNO_3_ concentration – 1 mM reaction period – ND^b^ reaction temperature – room temperature	SPR^a^ – 440 nm size – 12–200 nm shape – spherical	[32]
aqueous extract of *Ulva lactuca*	AgNO_3_ concentration – 1 mM reaction period – ND^b^ reaction temperature – ND^b^	SPR^a^ – 453 nm size – 20–50 nm shape – ND^b^	[56]

^a^SPR: surface plasmon resonance; ^b^ND: not defined.

Murugan and collaborators, in their study on the development of silver nanoparticles from aqueous extracts of *C. clavulatum* leaves, assessed that silver ions were reduced to form AgNPs. They indicated that the functional groups potentially involved in the reduction of silver ions were the amide and carbonyl groups of terpenoids and flavonoids [50].

Vinoth and colleagues also prepared AgNPs using brown seaweed, that is, the seaweed *S. polycystum* [51]. Initially, the authors prepared an aqueous extract (50 g of seaweed/500 mL H_2_O) via boiling for 30 min followed by cooling and filtration. The NPs were prepared from 10 mL of the aqueous extract filtrate with 90 mL of AgNO_3_ (1 mM). To increase the yield of silver nanoparticles, the sample was placed under magnetic stirring varying the heating temperatures (37–80 °C). The formation of NPs was verified from the color change in the solution to reddish brown. The possible chemical compounds evaluated as potential reducing agents in the biosynthesis of AgNPs were the secondary amines, aromatic primary amines, carboxylates, amides, alkenes, and aromatic compounds.

Roni and collaborators prepared AgNPs from red algae [52]. An aqueous extract of *H. musciformis* was obtained (10 g of seaweed leaves/100 mL of purified water) by heating the mixture for 5 min and decanting for 1 h. After this process, the mixture was filtered and stored for 5 days at 15 °C. Finally, the filtered solution was treated with an aqueous solution of AgNO_3_ (1 mM) and incubated at room temperature. The chemical compounds found were amino acid residues, aromatic rings, geminal methyls, ether linkages, flavones, terpenoids, aliphatic amines, and alcohols/phenols.

AgNPs were synthesized from an extract of the brown alga *S. natans* [53]*.* The extract was obtained via hot Soxhlet extraction of crushed leaves (40 °C) using ethanol, concentrated in a rotary vacuum evaporator, and finally stored at refrigerator temperature. A hydroalcoholic extract was produced by adding 1 mL of *S. natans* extract to 99 mL of purified water and 0.5 mL of Triton^®^. This extract was treated with AgNO_3_ (100 mM; 99:1) and conditioned at room temperature until the color changed to brown, indicative of the formation of AgNPs. Chemical analysis of the AgNPs demonstrated the presence of alcoholic compounds, phenolic compounds, aliphatic compounds, and carbonyl groups.

Green algae were also used to obtain AgNPs. Aziz et al., synthesized AgNPs from *U. lactuca* extract [56]. Initially, 10 g of the extract powder was extracted using a Soxhlet extractor (ethanol, 78 °C for 8 h) and concentrated in a rotary vacuum evaporator (40 °C). 100 mL of the extract was treated with AgNO_3_ solution (1 mM), showing the formation of NPs by the yellowish color. The authors did not report on the proportion volume ratio between extract and AgNO_3_ solution, nor the used part of the alga under study.

The red seaweed *G. firma* was used for the green synthesis of AgNPs [32]. The extract was prepared from ground seaweed. 10 g of the powder was added to purified water (100 mL) under boiling for 5 min. The filtrate was treated with aqueous AgNO_3_ solution (1 mM; the ratio of aqueous solution to AgNO_3_ solution was not mentioned) and incubated at room temperature. Finally, a yellowish-brown solution was observed, indicating the formation of AgNPs. Chemical analysis of the AgNPs demonstrated the presence of carbonyl groups from polyphenols such as catechin gallate, epicatechin gallate, epigallocatechin, epigallocatechin gallate, gallocatechin gallate, and flavin, amide groups, ethylene systems, and aliphatic amines/alcohols/phenols (polyphenols).

Gopu et al. also synthesized AgNPs from red algae [54]. *A. rigida* seaweed extract was prepared by adding pulverized seaweed (10 g) to 500 mL of purified water. The mixture was heated to a temperature of 80 °C under magnetic stirring for 20 min. Finally, the extract was filtered and centrifuged (12298*g* for 10 min). The NPs were obtained by mixing 10 mL of the extract supernatant with 90 mL of AgNO_3_ solution (1 mM) at a temperature of 37 °C, until a color change from colorless to reddish brown was observed. The chemical analysis of AgNPs demonstrated the presence of phenolic compounds, ether groups, and polysaccharides.

Also, blue algae were used to obtain silver nanoparticles. Elumalai et al. synthesized AgNPs from the aqueous extract of *O. sancta* [55]*.* A mixture of crushed seaweed (8 g) with purified water (100 mL) was heated to 60 °C for 20 min. The mixture was filtered to obtain the final extract, which was treated with AgNO_3_ (1 mM; 15:85 ratio) and incubated at 28 °C for 60 min.

**Figure 2:**
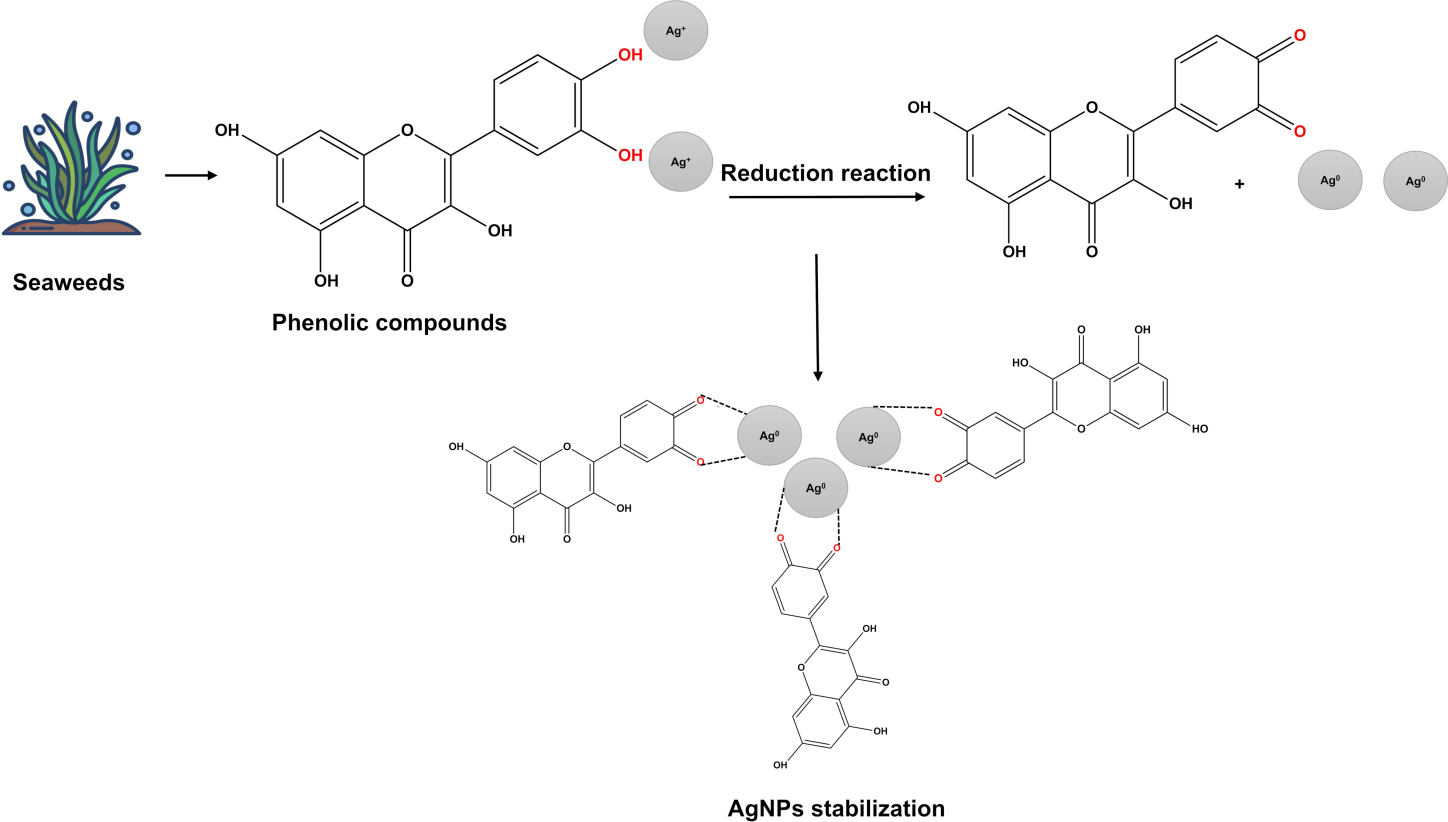
Green synthesis of silver nanoparticles (AgNPs) by seaweeds extracts. Created in BioRender. Rocha Formiga, F. (2024) https://BioRender.com/q62f029. This content is not subject to CC BY 4.0.

Composition of algae species, metal concentration, agitation, reaction time and temperature can impact the characteristics of AgNPs [57]. Thus, such systems must be well characterized, as discussed in the next section. Different chemical compounds are involved in the reduction of AgNO_3_ and the stabilization of AgNPs. Chemical analysis of AgNPs demonstrated the presence of alcohols, phenols, alkynes, aromatic compounds, long-chain fatty acids, secondary amides, and terpenoids. The predominance of phenolic compounds was evident in all species. These compounds act by reducing Ag^+^ ions to Ag^0^ and stabilize nanoparticles by capping [58] (Figure 2).

### Larvicidal activity of AgNPs against *Aedes aegypti*

*Aedes aegypti*, also known as the dengue mosquito, is a vector of important arboviruses, including Dengue, Zika, Chikungunya and Yellow Fever [6,59]. Among them, dengue fever is highlighted as this disease is endemic in more than 100 countries, proving to be an important public health problem. Its incidence has grown dramatically worldwide in recent decades, with cases reported to the WHO rising from 505,430 in 2000 to 5.2 million in 2019 [60]. Additionally, it is predicted that about 60 percent of the global population will be at risk of dengue in 2080 [61].

**Figure 3:**
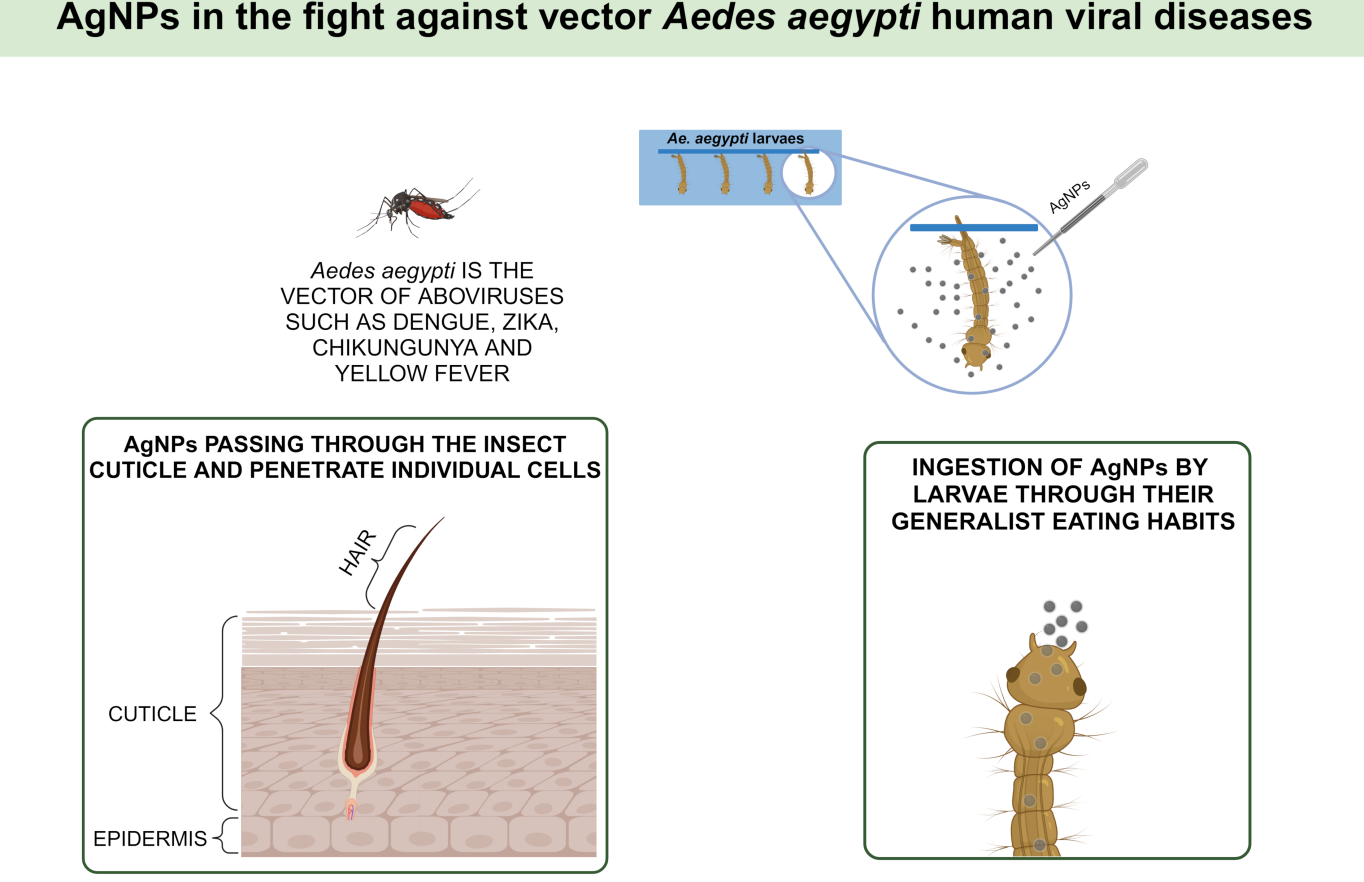
Potential of silver nanoparticles to be used in vector control against *Aedes aegypti*, according to [63]. Created in BioRender. Rocha Formiga, F. (2024) https://BioRender.com/y89s911. This content is not subject to CC BY 4.0.

Therefore, it is imperative to developed more advanced and efficient strategies for the control of mosquitoes and mosquito-borne diseases. Increased attention has been placed on using nanoparticles in controlling vector mosquitoes [62]. AgNPs synthesized from seaweed have been investigated as a vector control strategy based on their larvicidal properties. Table 2 summarizes data from bioassays with AgNPs synthesized from different species of seaweed against *A. aegypti* larvae. The mechanism of toxicity of AgNPs in mosquito larvae has recently been reported (Figure 3).

**Table 2:** Larvicidal activity of silver nanoparticles synthesized from seaweed against *Aedes aegypti*.

Algae	Exposure period (h)	Larval stage	LC_50_^a^ (µg/mL)	LC_90_^b^ (µg/mL)	References

*Sargassum polycystum*	24 48 72	L4	0.30 0.06 0.03	85.81 12.74 1.98	[51]
*Hypnea musciformi*s	24	L1–L3	ND^c^	ND^c^	[52]
*Sargassum natans*	ND^c^	L4	16.47	310.76	[53]
*Centroceras clavulatum*	ND^c^	L1–L4	21.460, 29.155	46.103–58.39	[50]
*Amphiroa rigida*	24	L3–L4	ND^c^	ND^c^	[54]
*Oscillatoria sancta*	24	L4	3.98	8.90	[55]
*Gracilaria firma*	24 48 72	ND^c^	ND^c^	ND^c^	[32]
*Ulva lactura*	ND^c^	L4	80.51	226.9	[56]

^a^Lethal concentration responsible for the mortality of 50% of individuals; ^b^lethal concentration responsible for the mortality of 90%; ^c^not defined.

The small size of AgNPs is linked to two pathways of action. First, AgNPs can pass through the insect cuticle and penetrate individual cells. The second way is the ingestion of AgNPs by larvae through their generalist eating habits. For both pathways, damage to the midgut, epithelial cells, and cortex in mosquito larvae can be observed, resulting in physiological changes such as shrinkage in the abdominal region, change in the shape of the thorax and loss of lateral hairs, oral brushes, and anal gills. These processes lead to oxidation and degradation of enzymes and organelles in the intracellular space of cells, affecting cellular physiological processes, leading to large-scale apoptosis and, consequently, larval death.

Vinoth, et al. [51] evaluated the larvicidal activity of AgNPs from *S. polycystum* seaweed extract against *A. aegypti* larvae. The L4 larvae were treated with 1 mL of NPs in 249 mL of distilled water. An increasing mortality was observed after periods of 24, 48, and 72 h, yielding the lowest LC_50_ value after a period of 72 h (Table 2). The response of the free extract against the larvae was not evaluated by the authors.

Similar results were observed in AgNPs developed by Roni and collaborators [52]. The authors evaluated the larvicidal activity after 24 h of exposure (25 larvae per 250 mL water) at different larval stages (L1–L4) of both the extract of *H. musciformis* (100–500 µg/mL) and the synthesized nanoparticles (10–50 µg/mL). The extract presented LC_50_ values ten times higher than the LC_50_ of NPs under the same conditions, showing the enhancement of larvicidal activity.

Another study presented data that corroborates the increase in larvicidal activity in AgNPs compared to the algae used for its synthesis. In this investigation, larvae (L4) were treated with the extract of the seaweed of *S. natans* (100–900 µg/mL) and with the synthesized NPs (100–300 µg/mL) [53]. The seaweed extract of *S. natans* showed an LC_50_ value of 299 µg/mL, while the derived AgNPs showed an LC_50_ value of 167 µg/mL. It is noteworthy that the treatment exposure time was not described by the authors.

Murugan et al. [50] were also able to obtain AgNPs with larvicidal activity derived from algae. The authors evaluated the larvicidal activity of seaweed extract of *C. clavulatums* (100–500 µg/mL) and the corresponding NPs (10–50 µg/mL) against *A. aegypti* larvae (L1–L4). The aqueous seaweed extract showed LC_50_ values of 269 µg/mL (L1), 310 µg/mL (L2), 348 µg/mL (L3), and 388 µg/mL (L4), while *C. clavulatum*-synthesized AgNPs were highly toxic against *A. aegypti,* revealing LC_50_ values of 21 µg/mL (L1), 24 µg/mL (L2), 26 µg/mL (L3), and 29 µg/mL (4). The treatment exposure time was not described by the authors.

Gopu, et al. synthesized and investigated the larvicidal potential of AgNPs from the seaweed extract *Amphiroa rigida* [54]. *Aedes aegypti* larvae at stages L3 and L4 were treated with *A. rigida* AgNPs (5–80 μg/mL). After 24 h of exposure, mortality of the larvae was observed above concentrations of 20 µg/mL (L3) and 40 μg/mL (L4). In this study, the LC_50_ values were not calculated; however, according to the mortality values presented, the LC_50_ values are in the range of 5–10 µg/mL for both larval stages. Furthermore, only the NPs were evaluated, and there is no mention of the larvicidal activity of the algae extract under study.

Elumai and colleagues evaluated the larvicidal activity of AgNPs derived from *Oscillatoria sancta* against larvae of *A. aegypti* [55]. Larvae in stages L3 and L4 were treated with the aqueous extract of the seaweed *Oscillatoria sancta* (10–100 µg/mL) and with AgNPs derived from *Oscillatoria sancta* (2–10 µg/mL); 24 h after treatment, the mortality of the larvae was evaluated. The NPs had a higher larvicidal activity than the seaweed aqueous extract, as also observed in other studies.

An increase in larvicidal activity was also observed in studies by Aziz [56]. An aqueous extract of the seaweed (100–900 µg/mL) and AgNPs (50–250 µg/mL) were applied to larvae in stage L4. The aqueous extract revealed LC_50_ values two times higher than those of NPs, demonstrating the enhancement of larvicidal activity. The authors did not highlight the period of time after which mortality was evaluated.

The formation of AgNPs after mixing the extracts with silver nitrate can be due to the synergy of biomolecules with reducing activity present in the extracts binding to the surface of the particles [64]. Despite the evident higher larvicidal activity of silver nanoparticles compared to algae extracts, there are significant variations in the results that must be considered. Larvicidal studies require standardization. Factors such as water volume, number of larvae, exposure time, larval stage, and mention of the presence or absence of larvae feeding must be established for better reliability of larvicidal studies. Furthermore, although the studies included did not carry out toxicity studies on non-target species, it is important to highlight the need for studies such as phytotoxicity, in vitro studies in cells, and in vivo models such as *Danio rerio* (zebrafish).

## Conclusion

Nanotechnology has great potential in current medicinal and agricultural systems, where pests and disease vectors are controlled by chemical pesticides that are toxic to non-target species and harmful to soil fertility and ecosystems. The biofabrication of metallic nanoparticles using marine resources has gained an exponential increase in attention over recent years. It is a promising area in nanoscience and nanotechnology that uses eco-friendly “green” methods. Silver nanoparticles (AgNPs) are known to have the benefits of being economical, energy efficient, and environmentally friendly.

The use of AgNPs synthesized from extracts of seaweed species against *Aedes aegypti* may be a viable option for replacing commercially available synthetic chemical insecticides, being able to surpass them in terms of larvicidal activity with lower toxicity to non-target organisms. Among the biogenic compounds of natural origin for green synthesis are flavonoids, tannins, terpenoids, saponins, phenols, and their derivatives. These compounds are responsible for the reduction and stabilization of silver nanoparticles.

The present review suggests that the green synthesis of nanomaterials from seaweed extracts is an environmentally friendly option for the control and prevention of vector-borne diseases. These nanomaterials are potential candidates for replacing commercially available toxic chemicals. Despite the proven formation of silver nanoparticles via green synthesis and their larvicidal activity against *A. aegypti*, some challenges still persist. Aspects such as the mechanism of action of AgNPs in the different stages of *A. aegypti*, resistance of mosquitoes to larvicides, and the long-term effects of NPs on non-target organisms still need to be elucidated to obtain a better understanding of their efficacy and safety.

## Data Availability

Data sharing is not applicable as no new data was generated or analyzed in this study.

## References

[1] Conway M J, Colpitts T M, Fikrig E (2014). Annu Rev Virol.

[2] Mansfield K L, Jizhou L, Phipps L P, Johnson N (2017). Front Cell Infect Microbiol.

[3] Alkan C, Bichaud L, de Lamballerie X, Alten B, Gould E A, Charrel R N (2013). Antiviral Res.

[4] Carpenter S, Groschup M H, Garros C, Felippe-Bauer M L, Purse B V (2013). Antiviral Res.

[5] Gomes H, de Jesus A G, Quaresma J A S (2023). One Health.

[6] de Santana Silva L L, Silva S C C, de Oliveira A P S, da Silva Nascimento J, de Oliveira Silva E, Coelho L C B B, Neto P J R, do Amaral Ferraz Navarro D M, Napoleão T H, Paiva P M G (2021). Acta Trop.

[7] Roiz D, Wilson A L, Scott T W, Fonseca D M, Jourdain F, Müller P, Velayudhan R, Corbel V (2018). PLoS Negl Trop Dis.

[8] Oliveros-Díaz A F, Pájaro-González Y, Cabrera-Barraza J, Hill C, Quiñones-Fletcher W, Olivero-Verbel J, Díaz Castillo F (2022). Arabian J Chem.

[9] Deka B, Babu A, Baruah C, Barthakur M (2021). Front Nutr.

[10] Bosly H A E-K, Salah N, Salama S A, Pashameah R A, Saeed A (2023). Acta Trop.

[11] Nasir S, Walters K F A, Pereira R M, Waris M, Ali Chatha A, Hayat M, Batool M (2022). J Asia-Pac Entomol.

[12] Porto A S, de Almeida I V, Vicentini V E P (2020). Rev Fitos.

[13] Vuitika L, Prates-Syed W A, Silva J D Q, Crema K P, Côrtes N, Lira A, Lima J B M, Camara N O S, Schimke L F, Cabral-Marques O (2022). Vaccines (Basel, Switz).

[14] Viana V C R, Machado F P, Esteves R, Duarte J A D, Enríquez J J S, Campaz M L M, Oliveira E E, Santos M G, Ricci-Junior E, Ruppelt B M (2023). Sustainable Chem Pharm.

[15] Chithiga A, Manimegalai K (2023). Exp Parasitol.

[16] Athanassiou C G, Kavallieratos N G, Benelli G, Losic D, Usha Rani P, Desneux N (2018). J Pest Sci.

[17] Benelli G, Caselli A, Canale A (2017). J King Saud Univ, Sci.

[18] Mikaili P, Maadirad S, Moloudizargari M, Aghajanshakeri S, Sarahroodi S (2013). Iran J Basic Med Sci.

[19] Shaalan E A-S, Canyon D, Younes M W F, Abdel-Wahab H, Mansour A-H (2005). Environ Int.

[20] Burin G R M, Formiga F R, Pires V C, Miranda J C, Barral A, Cabral-Albuquerque E C M, Vieira de Melo S A B, Braga M E M, de Sousa H C (2022). J Supercrit Fluids.

[21] Santhoshkumar J, Rajeshkumar S, Venkat Kumar S (2017). Biochem Biophys Rep.

[22] Klaus T, Joerger R, Olsson E, Granqvist C-G (1999). Proc Natl Acad Sci U S A.

[23] Galatage S T, Hebalkar A S, Dhobale S V, Mali O R, Kumbhar P S, Nikade S V, Killedar S G, Kumar S (2021). Silver Nanoparticles: Properties, Synthesis, Characterization, Applications and Future Trends. Silver Micro-Nanoparticles - Properties, Synthesis, Characterization, and Applications.

[24] Samuel M S, Ravikumar M, John J. A, Selvarajan E, Patel H, Chander P S, Soundarya J, Vuppala S, Balaji R, Chandrasekar N (2022). Catalysts.

[25] Ju-Nam Y, Lead J R (2008). Sci Total Environ.

[26] Bapat M S, Singh H, Shukla S K, Singh P P, Vo D-V N, Yadav A, Goyal A, Sharma A, Kumar D (2022). Chemosphere.

[27] Khan M, Khan M S A, Borah K K, Goswami Y, Hakeem K R, Chakrabartty I (2021). Environ Adv.

[28] Lazov L, Singh Ghalot R, Teirumnieks E, Samir K, Prabhat K, Chandra Shakher P (2021). Silver Nanoparticles - Preparation Methods and Anti-Bacterial/Viral Remedy Impacts against COVID 19. Silver Micro-Nanoparticles - Properties, Synthesis, Characterization, and Applications.

[29] Leema M, Sreekumar G, Sivan A, Pillai Z S (2019). Mater Today: Proc.

[30] Hassan A I, Samir A, Youssef H F, Mohamed S S, Asker M S, Mahmoud M G (2021). J Pharm Pharmacol (Chichester, U K).

[31] Sri Ramkumar S R, Sivakumar N, Selvakumar G, Selvankumar T, Sudhakar C, Ashokkumar B, Karthi S (2017). RSC Adv.

[32] Kalimuthu K, Panneerselvam C, Chou C, Lin S-M, Tseng L-C, Tsai K-H, Murugan K, Hwang J-S (2017). Hydrobiologia.

[33] Kalimuthu K, Cha B S, Kim S, Park K S (2020). Microchem J.

[34] Asmathunisha N, Kathiresan K (2013). Colloids Surf, B.

[35] Moorthi P V, Balasubramanian C, Mohan S (2015). Appl Biochem Biotechnol.

[36] Tamuly C, Hazarika M, Borah S C, Das M R, Boruah M P (2013). Colloids Surf, B.

[37] Hamedi S, Shojaosadati S A (2019). Polyhedron.

[38] Sajadi S M, Nasrollahzadeh M, Maham M (2016). J Colloid Interface Sci.

[39] Devi H S, Boda M A, Shah M A, Parveen S, Wani A H (2019). Green Process Synth.

[40] Ponnuchamy K, Jacob J A (2016). Nanotechnol Rev.

[41] Javan bakht Dalir S, Djahaniani H, Nabati F, Hekmati M (2020). Heliyon.

[42] Abdel-Raouf N, Al-Enazi N M, Ibraheem I B M (2017). Arabian J Chem.

[43] Abdel-Raouf N, Al-Enazi N M, Ibraheem I B M, Alharbi R M, Alkhulaifi M M (2019). Saudi J Biol Sci.

[44] Roseline T A, Murugan M, Sudhakar M P, Arunkumar K (2019). Environ Technol Innovation.

[45] Gnanadesigan M, Anand M, Ravikumar S, Maruthupandy M, Syed Ali M, Vijayakumar V, Kumaraguru A K (2012). Appl Nanosci.

[46] Davis T A, Volesky B, Mucci A (2003). Water Res.

[47] Selvaraj P, Neethu E, Rathika P, Jayaseeli J P R, Jermy B R, AbdulAzeez S, Borgio J F, Dhas T S (2020). Biocatal Agric Biotechnol.

[48] Gurunathan S, Raman J, Abd Malek S N, John P A, Vikineswary S (2013). Int J Nanomed.

[49] Dadashpour M, Firouzi-Amandi A, Pourhassan-Moghaddam M, Maleki M J, Soozangar N, Jeddi F, Nouri M, Zarghami N, Pilehvar-Soltanahmadi Y (2018). Mater Sci Eng, C.

[50] Murugan K, Aruna P, Panneerselvam C, Madhiyazhagan P, Paulpandi M, Subramaniam J, Rajaganesh R, Wei H, Alsalhi M S, Devanesan S (2016). Parasitol Res.

[51] Vinoth S, Shankar S G, Gurusaravanan P, Janani B, Devi J K (2019). J Cluster Sci.

[52] Roni M, Murugan K, Panneerselvam C, Subramaniam J, Nicoletti M, Madhiyazhagan P, Dinesh D, Suresh U, Khater H F, Wei H (2015). Ecotoxicol Environ Saf.

[53] Barnawi A, Tariq SAlghamdi T, Mahyoub J, Al-Ghamdi K J (2019). Entomol Zool Stud.

[54] Gopu M, Kumar P, Selvankumar T, Senthilkumar B, Sudhakar C, Govarthanan M, Selva Kumar R, Selvam K (2021). Bioprocess Biosyst Eng.

[55] Elumalai D, Hemavathi M, Rekha G S, Pushpalatha M, Leelavathy R, Vignesh A, Ashok K, Babu M (2021). Sens Bio-Sens Res.

[56] Aziz A T (2022). IET Nanobiotechnol.

[57] Srikar S K, Giri D D, Pal D B, Mishra P K, Upadhyay S N (2016). Green Sustainable Chem.

[58] Omidi S, Sedaghat S, Tahvildari K, Derakhshi P, Motiee F (2018). Green Chem Lett Rev.

[59] Liu Y, Lillepold K, Semenza J C, Tozan Y, Quam M B M, Rocklöv J (2020). Environ Res.

[60] WHO World Health Organization Dengue And Severe Dengue.

[61] Messina J P, Brady O J, Golding N, Kraemer M U G, Wint G R W, Ray S E, Pigott D M, Shearer F M, Johnson K, Earl L (2019). Nat Microbiol.

[62] Gunathilaka U M T M, de Silva W A P P, Dunuweera S P, Rajapakse R M G (2021). RSC Adv.

[63] Rodrigues dos Santos D, Lopes Chaves L, Couto Pires V, Soares Rodrigues J, Alves Siqueira de Assunção M, Bezerra Faierstein G, Gomes Barbosa Neto A, de Souza Rebouças J, Christine de Magalhães Cabral Albuquerque E, Alexandre Beisl Vieira de Melo S (2023). Int J Pharm.

[64] Borase H P, Patil C D, Salunkhe R B, Narkhede C P, Salunke B K, Patil S V (2013). J Nanomed Biother Discovery.

